# Two-Dimensional Fluorescence Difference Spectroscopy of ZnO and Mg Composites in the Detection of Physiological Protein and RNA Interactions

**DOI:** 10.3390/ma10121430

**Published:** 2017-12-15

**Authors:** Amanda Hoffman, Xiaotong Wu, Jianjie Wang, Amanda Brodeur, Rintu Thomas, Ravindra Thakkar, Halena Hadi, Garry P. Glaspell, Molly Duszynski, Adam Wanekaya, Robert K. DeLong

**Affiliations:** 1 Department of Anatomy and Physiology, Nanotechnology Innovation Center of Kansas State (NICKS), Kansas State University, Manhattan, KS 66506, USA; arhoffman@ksu.edu (A.H.); maggiewu1992@vet.k-state.edu (X.W.); ravithakkar@vet.k-state.edu (R.T.); 2 Department of Biomedical Science, Missouri State University, Springfield, MO 65897, USA; JWang@MissouriState.edu (J.W.); ABrodeur@MissouriState.edu (A.B.); rtthomas3@uh.edu (R.T.); 3 University of Notre Dame, Notre Dame, IN 46556, USA; hhadi@nd.edu; 4 Department of Chemistry, Virginia Commonwealth University, Richmond, VA 23284, USA; Garry.P.Glaspell@erdc.dren.mil; 5 Department of Chemistry, Missouri State University, Springfield, MO 65897, USA; Duszynski314@live.missouristate.edu (M.D.); Wanekaya@MissouriState.edu (A.W.)

**Keywords:** zinc oxide, nanocomposites, aptamer, thrombin, angiotensin-converting enzyme, ribonuclease A, RNA, two-dimensional fluorescence difference spectroscopy

## Abstract

Two-dimensional fluorescence difference spectroscopy (2-D FDS) was used to determine the unique spectral signatures of zinc oxide (ZnO), magnesium oxide (MgO), and 5% magnesium zinc oxide nanocomposite (5% Mg/ZnO) and was then used to demonstrate the change in spectral signature that occurs when physiologically important proteins, such as angiotensin-converting enzyme (ACE) and ribonuclease A (RNase A), interact with ZnO nanoparticles (NPs). When RNase A is bound to 5% Mg/ZnO, the intensity is quenched, while the intensity is magnified and a significant shift is seen when torula yeast RNA (TYRNA) is bound to RNase A and 5% Mg/ZnO. The intensity of 5% Mg/ZnO is quenched also when thrombin and thrombin aptamer are bound to the nanocomposite. These data indicate that RNA–protein interaction can occur unimpeded on the surface of NPs, which was confirmed by gel electrophoresis, and importantly that the change in fluorescence excitation, emission, and intensity shown by 2-D FDS may indicate specificity of biomolecular interactions.

## 1. Introduction

Metamaterials or composites combine the advantages of multiple elements in the nanoscale and have unique physico-chemical properties [1,2,3]. There is currently a great deal of interest in nanobio sensors where many of these applications involve zinc oxide (ZnO) nanoparticles (NPs) or doped derivatives [4,5,6,7,8,9,10,11,12,13,14]. A variety of different target molecules, including but not limited to riboflavin, mucin-1, bisphenol A, ATP, acetamiprid, micro-RNA, thrombin, and different types of cancer cells (Hela, SK-BR-3, K562), have been detected [4,5,6,7,8,9,10,11,12,13,14]. ZnO has been fabricated into a variety of different structures with various other components including carbon quantum dots [4], platinum [5], AlGaN [6], Au (gold) [7,8,11], Co (cobalt) [9], and graphene [12]. In many cases, detection by these nanocomposite sensors is based on electrochemistry or photoelectrochemistry [5,7,9,11,12,14]. However, in some cases, detection has been based on electrochemiluminescence [4], field effect transistors (FET) [13], or 3-D quantitative fluorescence imaging [6].

Our group has recently reported that two-dimensional fluorescence difference spectroscopy (2-D FDS) can be used as a new characterization technique for nanomaterials and can be used to probe the nanobio interface [15]. This method has an advantage over the other methods of detection listed above because it does not rely on a dye or fluorophore to detect the presence of nanomaterials and their interactions, and it is a simple technique that does not require complex analysis [15]. We noted that this technique is sensitive to the nanoparticle synthetic method and composition and that a spectral shift in two dimensions occurs upon either RNA or protein interactions at the surface [15,16]. Here, a fluorescence quenching or shift occurs via energy transfer or via overlap between the biomolecule and the nanomaterial. We demonstrate that 2-D FDS can provide unique spectral signature of composite NPs and, further, can be utilized to determine RNA and protein interaction specificity.

## 2. Results

### 2.1. Iteration of 2-D FDS in the Analysis of Nanoparticle Composition

**Figure 1:**
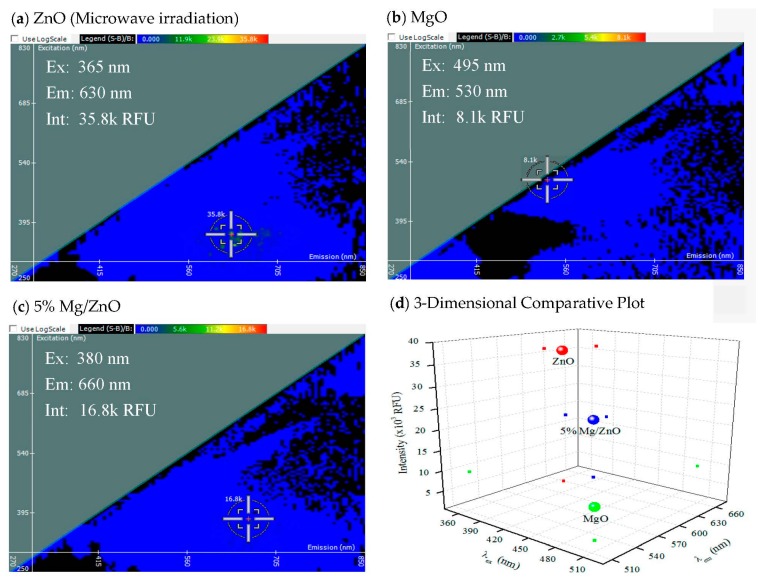
2-D FDS of (**a**) zinc oxide nanoparticles (NPs) synthesized by microwave irradiation; (**b**) magnesium oxide nanoparticles; and (**c**) 5% magnesium with 95% zinc oxide; (**d**) Three-dimensional comparative plot of the three nanocomposites.

Synthesis of the 5% Mg/ZnO composite was compared to the pure parent material (Figure 1).

**Figure 2:**
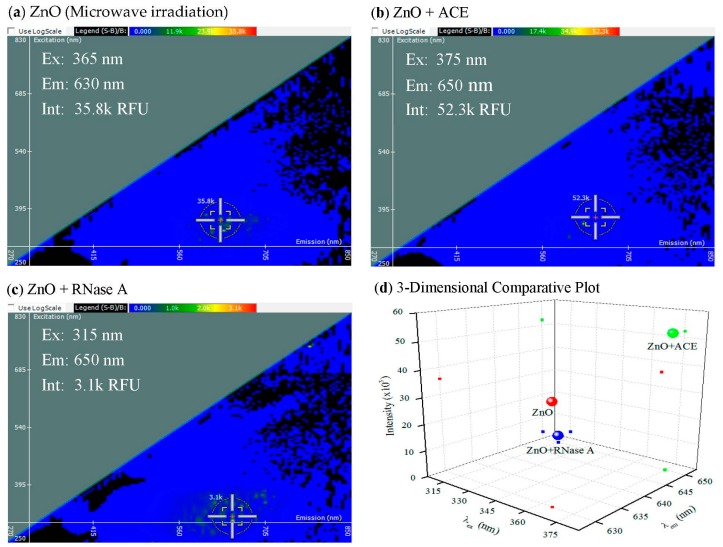
2-D FDS of (**a**) ZnO nanoparticles synthesized by microwave irradiation; (**b**) ACE bound to microwave irradiation ZnO nanoparticles; and (**c**) ribonuclease A (RNase A) bound to microwave irradiation ZnO nanoparticles; The (**d**) 3-dimensional comparative plot of the three ZnO nanoparticle interactions.

**Figure 3:**
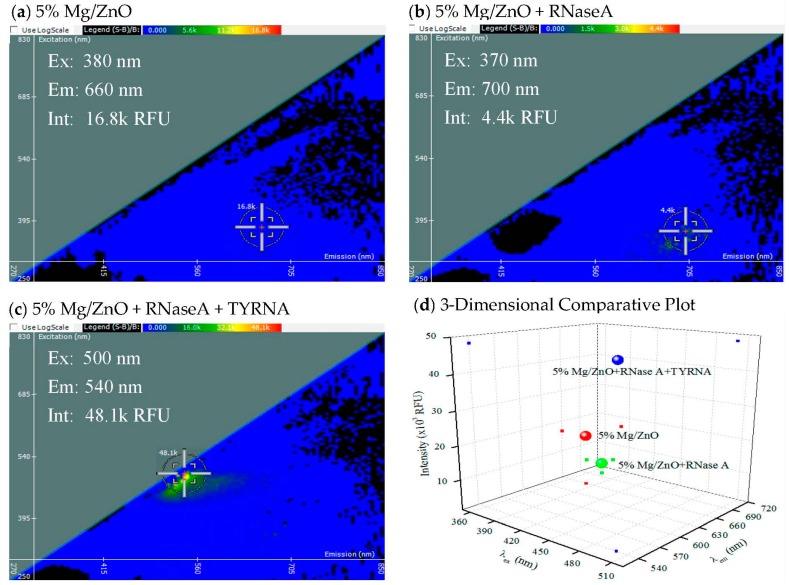
2-D FDS of (**a**) 5% magnesium with 95% zinc oxide; (**b**) RNase A bound to 5% Mg/ZnO; and (**c**) torula yeast RNA and RNase A bound to 5% Mg/ZnO; (**d**) Three-dimensional comparative plot of the three 5% Mg/ZnO RNase A interactions.

**Figure 4:**
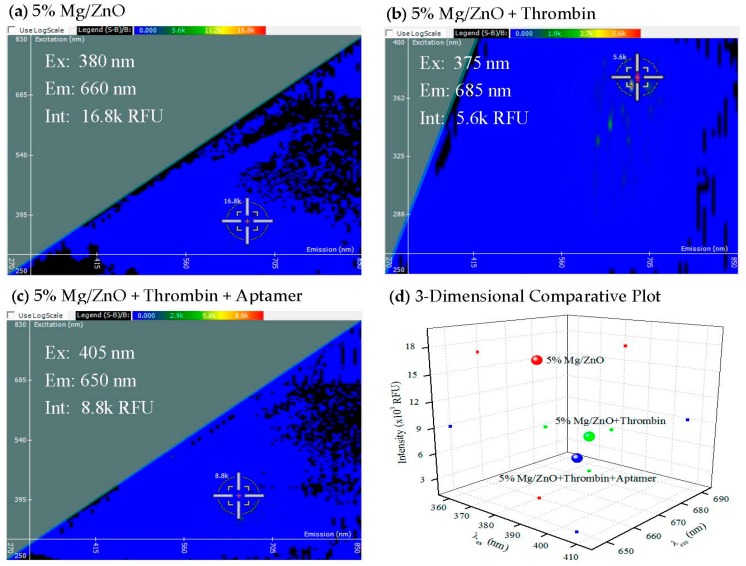
2-D FDS of (**a**) 5% magnesium with 95% zinc oxide; (**b**) thrombin bound to 5% Mg/ZnO; and (**c**) thrombin and thrombin aptamer bound to 5% Mg/ZnO; (**d**) Three-dimensional comparative plot of the three 5% Mg/ZnO thrombin interactions.

In Figure 1, the spectral signatures of three different nanomaterials are compared. ZnO, MgO, or the composite excitation and emission spectrum is shown in 2-D in Panels a, b, and c, respectively. The *y*-axis represents the excitation wavelength in nm, the *x*-axis represents the emission wavelength in nm, and the scale above the 2-D FDS plot represents the intensity in relative fluorescence units (RFU). Each spectral signature is unique to the nanomaterial, as shown by the 3-D graph where the intensity of the composite was midway between ZnO (highest) and MgO (lowest), as shown in Panel d. The *x*-axis denotes excitation wavelength in nm, the *y*-axis denotes emission wavelength in nm, and the *z*-axis denotes intensity in RFU. The large spheres in Panel d denote the intersection of the excitation, emission, and intensity values of each nanomaterial as labeled. The small dots show the individual values for excitation, emission, and intensity for simple reading of the 3-D graph, and these values are applied to the following figures (Figure 1, Figure 2, Figure 3 and Figure 4). The 2-D FDS method can be used to distinguish between different nanomaterials. This also demonstrates the nature of nanocomposites, where certain properties of each nanoparticle are combined to form new properties. The fluorescent intensity of ZnO is 35.8 k RFU, while the fluorescent intensity of MgO is 8.1 k RFU. When they are combined to form 5% Mg/ZnO, the fluorescent intensity is in between the two nanoparticles at 16.8 k RFU.

### 2.2. Iteration of 2-D FDS in the Analysis of Nanoparticle Interaction to Protein

The binding events to angiotensin-converting enzyme (ACE) or ribonuclease A (RNase A), two physiologically relevant proteins, were characterized by 2-D FDS (Figure 2).

In Figure 2, the spectral signature of ZnO is compared to the spectral signatures of ZnO with either ACE or RNase A bound to it, and it is seen that the interaction of protein with ZnO causes three shifts, in the excitation wavelength, emission wavelength, and the fluorescence intensity. The same shift occurs in the emission when either ACE or RNase A is bound to ZnO. There is a shift in the excitation as well, but there is a significant difference between the ACE and RNase A excitation wavelengths. There is also a significant change in the intensities when ACE or RNase A are bound to ZnO. The intensity of ZnO is 35.8 k RFU, and this value is quenched to 3.1 k RFU when RNase A is bound to ZnO. Binding RNase A to ZnO has the opposite effect and magnifies the intensity to 52.3 k RFU. This is likely due to different orientations of the proteins at the surface of the NPs and whether interaction exposes fluorescently active amino acids (e.g., tryptophan), hence resulting in fluorescent quenching or activation (unpublished observations).

### 2.3. Iteration of 2-D FDS in the Analysis of Protein–RNA Interaction to the Nanoparticle

In cells and tissues, magnesium (Mg) is well-known to mediate protein and especially RNA structure and stability. Hence, we hypothesized that the incorporation of Mg into ZnO NPs may allow for interaction and that, for an RNA binding protein (RNase A), the addition of RNA (from torula yeast) may exchange RNA at the surface as a function of binding to protein and that this signal might be detected by 2-D FDS. These data are shown next (Figure 3).

In Figure 3, the spectral signature of 5% Mg/ZnO is compared to the spectral signatures of RNase A, as well as both TYRNA and RNase A, bound to 5% Mg/ZnO. As expected, the data reveal a shift in the emission during both interactions, but in opposite directions. There is a slight shift in the excitation when RNase A only is bound to 5% Mg/ZnO, and a significant excitation shift when RNase A is bound followed by the addition of TYRNA. The fluorescent intensity of 5% Mg/ZnO is quenched when RNase A is bound to it, changing from a value of 16.8 to 4.4 k RFU. The fluorescent intensity of 5% Mg/ZnO is significantly magnified when TYRNA and RNase A are bound to it, with an intensity of 48.1 k RFU. As mentioned previously, this is likely explained by the addition of RNA to RNase, causing conformational change allowing exposure of the protein’s own fluorescence moieties and some energy transfer between it, the RNA molecule, and NP surface.

### 2.4. Iteration of 2-D FDS in the Detection of Specific Aptamer–Protein Interaction

To detect aptamer target interaction, the classic and perhaps most well characterized thrombin RNA aptamer system [17,18] was employed in conjunction with the 5% Mg/ZnO NP composite (Figure 4).

In Figure 4, the spectral signature of 5% Mg/ZnO is compared with the spectral signatures of 5% Mg/ZnO bound to either thrombin or both thrombin aptamer and thrombin. When thrombin is bound to 5% Mg/ZnO, there is a shift in emission and a slight change in excitation. A quenching effect is also created, with the fluorescent intensity shifting from 16.8 to 5.6 k RFU. Binding thrombin aptamer and thrombin to 5% Mg/ZnO creates a shift in the emission and a larger shift in excitation. The fluorescent intensity is quenched to a value of 8.8 k RFU as well. These results are interpreted as a strong association of the aptamer/target protein at the surface quenching fluorescence.

### 2.5. NPs Do Not Affect Specific Aptamer–Protein Interaction by Gel Mobility Shift

**Figure 5:**
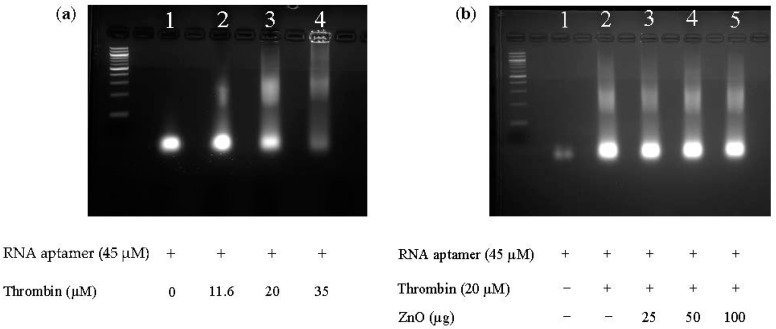
Electrophoretic mobility shift assay (EMSA) of (**a**) RNA aptamer with varying concentrations of thrombin and (**b**) RNA aptamer with or without thrombin and with varying concentrations of ZnO.

To confirm that the NPs do not interfere with aptamer–target interactions, we employed a classical electrophoretic mobility shift assay (EMSA). In this experiment, an increasing amount of thrombin was added to the interaction tube and was titrated with or without the addition of NPs. NP–protein interactions have been well documented by researchers in our lab and by others [19,20], making it possible to show that the introduction of the thrombin aptamer after the thrombin protein was bound resulted in total fluorescence quenching, as shown previously above, and that the results of the electrophoretic mobility shift assay (EMSA) shows this by the decreasing fluorescent intensity of the bands in Figure 5a as the thrombin concentration increases.

In Figure 5, binding of thrombin to RNA aptamer is detected by EMSA and is concentration-dependent (Panel a). The ladder fragments of the 25 nucleotide Toggle-25t RNA aptamer phosphothioate is denoted by “25 NT” under the ladder. In Figure 5b, the band intensity in Lane 1 is lower than the band intensity in Lane 1 of Figure 5a. This is caused by gel-to-gel staining variability, and shown in the figure due to the gel imager automatically correcting fluorescence, leading to different band intensities. Introduction of the NP, ZnO, was used in this case because of its protein binding affinity [19,20] and was shown not to disrupt the specific biomolecular interaction, if anything leading to fewer non-specific aggregates (staining in the well) and stabilizing the complex at all concentrations tested.

## 3. Discussion

In summary, 2-D FDS was used to assess the fluorescence of composite materials (5% Mg/ZnO) and their interaction with RNA and protein. Fluorescence excitation occurs when electrons absorb energy to an excited state; and, when it relaxes to the ground state, fluorescent emission results. Fluorescent quenching occurs when electron clouds of nanomaterials interact with each other at the van der Waals radii, and energy is dissipated as heat when the excited electron moves back to the ground state [21]. We postulate that the binding of a biomolecule, such as RNA or protein, onto the surface of a nanomaterial with similar electronic fluorescence properties will yield a quenching or fluorescent shift effect. This technique can detect the presence of physiologically relevant proteins such as angiotensin-converting enzyme (ACE), ribonuclease A (RNase A), and thrombin, as well as RNA such as that derived from torula yeast (TYRNA) or RNA aptamer. Using the thrombin–aptamer system, we have shown that 2-D FDS can be used to detect specific RNA–protein interaction, where the changes in fluorescence are unique in comparison to the TYRNA–RNase system. These findings imply that 2-D FDS in conjunction with nanomaterial composites may have ramifications in the detection of protein, RNA, and their interactions and may be a useful detection platform for analytical assays more generally, aiding and supporting classical methods such as EMSA.

## 4. Materials and Methods

### 4.1. Materials

Mg/ZnO with a 5% concentration was provided by Dr. A. Wanekaya (Missouri State University, Springfield, MO, USA) and synthesized via hydrothermal methods. ZnO nanoparticles synthesized by microwave irradiation methods were obtained from Dr. G. Glaspell (Virginia Commonwealth University, Richmond, VA, USA). MgO was purchased from Sigma-Aldrich (St. Louis, MO, USA). HPLC grade water was purchased from Acros Organics (Belgium, WI, USA). Angiotensin-converting enzyme from rabbit lung (ACE), ribonucleic acid from torula yeast (TYRNA), and thrombin were obtained from Sigma-Aldrich. Thrombin aptamer was obtained from Trilink Biotechnology (San Diego, CA, USA). RNase A was purchased from Thermo Fisher Scientific (Waltham, MA USA). A black 96-well microplate was purchased from Midsci Corp. (Valley Park, MO, USA).

### 4.2. Stock Preparation

Using the Mettler Toledo Excellence XS Analytical Balance (Mettler-Toledo, LLC., Columbus, OH, USA), nanomaterials were weighed out at 3 mg and suspended in 1 mL of HPLC grade water. The nanomaterial solution is then dispersed for 5 min with 20 s pulses at 50% amplitude using Sonics Vibracell VCX 130 probe ultra-sonicator (Sonics & Materials, Inc., Newtown, CT, USA).

### 4.3. Nanomaterial Alone

For the 2-D FDS of each nanomaterial on its own, 66.7 μL of the dispersed nanomaterial solution was added to a sterile microcentrifuge tube with 133.3 μL of HPLC grade water and then re-suspended, giving a final nanomaterial concentration of 1 mg/mL. This solution was then added to a well in a black 96-well microplate and read by the Molecular Devices Spectramax i3x spectrophotometer (Molecular Devices, LLC., Sunnyvale, CA, USA).

### 4.4. Protein Interactions

For the protein interaction tests, 1 mg/mL of 5% Mg/ZnO was incubated on ice with 0.1 mg/mL thrombin or RNase A for 30 min on the orbital shaker at 150 rpm, then transferred to a well of a black 96-well microplate and read by the Molecular Device Spectramax i3x spectrophotometer. For the specificity tests, the procedure above was followed, and 0.1 mg/mL thrombin aptamer or TYRNA were added to their respective solutions, incubated on ice again under the same conditions, and then transferred to a microplate and read by the spectrophotometer. The procedure above was followed for the ZnO tests with 0.038 mg/mL ACE or RNase A added instead.

### 4.5. Molecular Device Settings

The Molecular Devices Spectramax i3x spectrophotometer utilizes the Spectral Optimization Wizard that is included in the Softmax Pro 6.4.2 accompaniment software (Sunnyvale, CA, USA) to scan the black 96-well microplate without the lid. The device reads the fluorescence endpoints of unknown wavelengths. The photomultiplier (PMT) gain was high, flashes per read was six, and wavelength increment was 5 nm. The microplate was shaken in a linear mode at medium intensity before the first read and was read from the top at a height of 1 mm. The range of excitation and emission wavelengths was 250–830 nm and 270–850 nm, respectively. The previously observed range in emission values of metal oxides is 25 nm [15].

### 4.6. RNA Aptamer-Thrombin and Gel Mobility Shift

Binding specificity of Toggle-25t RNA aptamer phosphothioate (5’-GGG AAC AAA GCU GAA GUA CUU ACC C-3’) (Integrated DNA Technology, Reference number: 118200374, Coralville, IA, USA) to varying concentrations of human alpha thrombin (Enzyme Research Laboratories, Catalog number: HT 1002a, Pittsburgh, PA, USA) was observed using agarose gel electrophoresis. The lyophilized RNA aptamer was suspended in diethyl pyrocarbonate (DEPC) water at a final concentration of 163 μM. Human alpha thrombin at a concentration of 3.41 mg/mL was obtained in buffer solution containing 50 mM sodium citrate, 0.2 M NaCl, and 0.1% polyethylene glycol (PEG) (pH 6.5). All reaction samples in 20 μL was composed of 45 μM RNA aptamer in HBS buffer containing 150 mM NaCl, 2 mM CaCl_2_, 20 mM HEPES (pH = 7.35 ± 0.1), and 6.25 mM MgCl_2_. With the exception of control (RNA aptamer alone), the rest of the reaction mixtures were titrated with protein alpha thrombin with increasing concentrations (11.61, 20, and 35 μM) to achieve aptamer/protein molar ratios as indicated: 1:0.25, 1:0.50, and 1:0.7. ZnO nanoparticles were washed with water three times and then with ethanol with concentrations of 50% (v/v), 70% (v/v), and 95% (v/v) three times. Colloidal suspension of ZnO nanoparticles was prepared by ultrasonication (Fischer Scientific, Model number: FS20, Pittsburgh, PA, USA) for 45 min at a concentration of 1 mg/mL in water. Reaction samples of RNA aptamer–thrombin–ZnO nanoparticles in 20 μL contained 6.25 mM MgCl_2_, 45 μM RNA aptamer, 20 μM alpha-thrombin, and ZnO colloidal suspension at desired concentrations in HBS buffer. The samples were incubated at room temperature for 30 min and then at 37 °C for 10 min prior to gel electrophoresis analysis. Following incubation, the reaction mixture was loaded on to a 3% (w/v) agarose gel containing 0.005% (v/v) ethidium bromide. The agarose gel was electrophoresed at room temperature (approximately 25 °C) in 1 X TAE containing 40 mM Tris (pH = 7.6), 20 mM acetic acid, and 1 mM EDTA for 45 min at 110 V (Bulletin 6205). Agarose gels were imaged using EL Logic 200 imaging system (Eastman Kodak Company, Rochester, NY, USA) and quantified using Kodak Image Pro software. Each of the experiments were replicated three times and the quantification results were imported into Excel, where the mean, standard deviation, and error for the data sets were calculated.

## 5. Conclusions

We used two-dimensional fluorescence difference spectroscopy to study ZnO and Mg composites and their interactions with physiological proteins and RNA.
